# Analysis of the disbandment of elite ice hockey teams in Korea and measures for improvement

**DOI:** 10.3389/fspor.2025.1517277

**Published:** 2025-03-19

**Authors:** Young Man Park, Mi Ae Shin, Dong Hyun Won

**Affiliations:** ^1^ Department of Physical Education, Korea National Sport University, Seoul City, Republic of Korea; ^2^ Department of Human Care Lifelong Education, Daejin University, Pocheon City, Gyeonggi-do, Republic of Korea

**Keywords:** elite sports, ice hockey, disbandment, sports team, phenomenon analysis

## Abstract

**Background:**

As Korea's elite ice hockey teams continue to disband, the elite ice hockey environment continues to deteriorate. Therefore, the purpose of this study is to analyze the phenomenon, identify problems, and suggest improvement measures.

**Methods:**

The Delphi technique was conducted in three rounds with the nine research participants who had experience with team dissolution.

**Results:**

The de-tailed items were identified by the categories of coaches, players, and parents, and the coaches' detailed items were “lack of coaches” college entrance exam solving capabilities', “student assault incidents committed by coaches”, and “poor working conditions and treatment of coaches”. The detailed items for players were “lack of basic knowledge of team sports”, “lack of basic manners in team sports”, “decline in teamwork due to individual egoism”, and “perception that parents” opinions are prioritized over coaches'. The detailed items for parents were “parental involvement in training”, “parental involvement in hiring coaches”, “parental involvement in player management”, “parental involvement in school operation”, and “parental involvement in game participation”.

**Conclusions:**

Based on the problems and discussions that emerged from this study, if we conclude, coaches, players, and parents should recognize that each of them has their own problems, rather than blaming each other, and in the actual field, practical efforts for improvement should be reflected at the team level. In particular, an atmosphere in which parents truly want to change should be created, and in order to improve in the long term, efforts should be made based on academic data.

## 1 Introduction

Until 2016, the Korea Sports Council based on the National Sports Promotion Act and the Korea Council of Sport for All based on the Civil Act had been in charge of the promotion of professional sports and sports for all in Korea (1). In accordance with Article 33 of the National Sports Promotion Act amended in March 2015, the Korea Sports Council and the Korea Council of Sport for All were integrated, and strengthening the link between professional sports and sports for all was expected to lead to strengthening the foundation for finding and nurturing new and excellent players and activation of sports for all (2).

**Figure 1:**
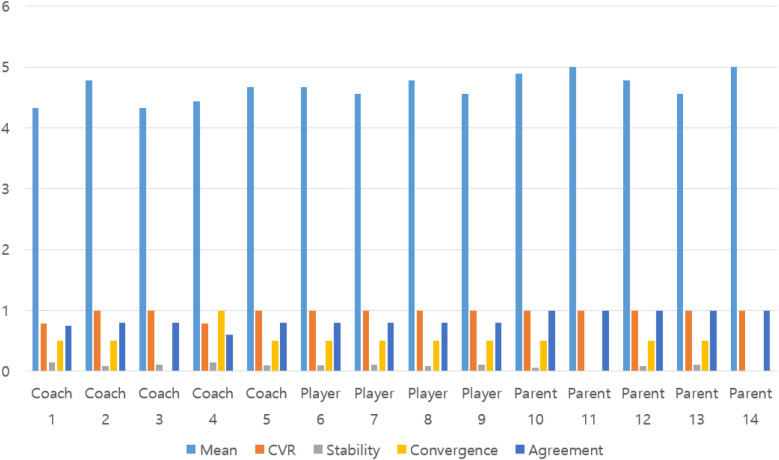
Results of 3rd delphi survey.

**Table 1:** Detailed composition of the expert group.

Classification	Detailed career	Professional experience	Current position
Expert A	Department of Physical Education	10 years or more	Professor
Expert B	Scholar majoring in Sports Industry and Management	15 years or more	Doctoral-level researcher
Expert C	Scholar majoring in Sports Industry and Management	15 years or more	Doctoral-level researcher

**Table 2:** Composition of research participants.

Target	Related career	Sex	Group	Alma mater team disbanded/not disbanded
Participant 1	10 years or more	Male	Director/Coach	Middle and high school teams disbanded
Participant 2	10 years or more	Male	Director/Coach	Middle school team disbanded
Participant 3	10 years or more	Male	Director/Coach	High school and university teams disbanded
Participant 4	10 years or more	Male	Director/Coach	High school team to be disbanded
Participant 5	10 years or more	Male	Director/Coach	University team disbanded
Participant 6	—	Female	Parents	Middle school team disbanded
Participant 7	—	Female	Parents	High school and university teams disbanded
Participant 8	—	Female	Parents	High school and university teams disbanded
Participant 9	—	Male	Parents	High school and university teams disbanded

**Table 3:** Research procedures.

Classification	Round 1	Round 2	Round 3
Questionnaire	Semi-structured	Structured (agree/disagree)	Structured (Likert 5 points)
Survey details	Analysis of the team disbandment in elite ice hockey	Verification of consensus and importance among experts on the problem items identified as the cause of team disbandment	
Analysis items	Expert verification	Classification and verification of majority and minority opinions on Round 1 analysis items	Verification of mean, CVR, stability, convergence, and agreement

**Table 4:** Operating status of elite ice hockey team.

Classification	School name	Region	Type	Remarks
Middle school	Gyeongseong	Seoul	Private	
	Kyunghee	Seoul	Private	
	Gwangwoon	Seoul	Private	
	Geunmyeong	Gyeonggi-do	Private	
	Namchuncheon	Gangwon-do	Public	Female team
	Bundang	Gyeonggi-do	Public	
	Jungdong	Seoul	Private	
High school	Gyeonggi	Seoul	Public	
	Gyeongbok	Seoul	Public	
	Gyeongseong	Seoul	Private	
	Gwangseong	Seoul	Private	
	Jungdong	Seoul	Private	Scheduled to be disbanded in 2025
University	Kyunghee	Gyeonggi-do	Private	Maintaining existence
	Korea	Seoul	Private	Ice rink on campus
	Kwangwoon	Seoul	Private	Ice rink on campus
	Yonsei	Seoul	Private	
Works team	HL Anyang	Gyeonggi-do	HL	
	Suwon City Hall	Gyeonggi-do	Suwon	

**Table 5:** Registration status of elite ice hockey players.

Classification	School name	Number of people
Middle school	Gyeongseong	21
	Kyunghee	26
	Gwangwoon	19
	Geunmyeong	25
	Namchuncheon	13
	Bundang	23
	Jungdong	24
Middle School Subtotal		151
High school	Gyeonggi	23
	Gyeongbok	20
	Gyeongseong	21
	Gwangseong	22
	Jungdong	16
High School Subtotal		102
University	Kyunghee	15
	Korea	27
	Kwangwoon	25
	Yonsei	25
University Subtotal		92
Works team	HL Anyang	23
	Suwon City Hall	12
Works Team subtotal		35
Total		380

**Table 6:** Status of disbanded elite ice hockey teams.

Classification	School name	Region	Type	Disbandment period
Middle school	Gyeongmin	Gyeonggi-do	Private	2015
	Gwangseong	Seoul	Private	2023
	Boseong	Seoul	Private	—
	Yeonseong	Incheon	Private	2015
High school	Shinsong	Incheon	Public	2015
	Seondeok	Seoul	Public	2016
	Boseong	Seoul	Private	2021
University	Hanyang	Gyeonggi-do	Private	2021
Works team	Daemyung Killer Whales	Seoul	Daemyung Sono Group	2021
	Korea Armed Forces Athletic Corps Sangmu	Gyeongbuk	Ministry of Defense	2019
	High1	Gangwon-do	High1 Resort	2023

**Table 7:** Results of the 1st Delphi survey.

NO	Derived items
1	Lack of basic knowledge about team sports
2	Lack of basic manners in team sports
3	Decrease in teamwork due to individual egoism
4	Lack of leadership capacity of coaches
5	Lack of leadership capacity of coaches in solving university entrance exams
6	Awareness of prioritizing parents’ opinions over those of coaches
7	Parental involvement in training
8	Parental involvement in hiring coaches
9	Student violence committed by coaches
10	Parental involvement in player management
11	Lack of awareness of what educators should have
12	Parental involvement in school operation
13	Poor working conditions and treatment of coaches
14	Parental involvement in game participation

**Table 8:** Results of 2nd Delphi survey.

Category		NO	Derived items
Majority opinions	Coach	4	Lack of leadership capacity of coaches
		5	Lack of leadership capacity of coaches in solving university entrance exam problem
		9	Student violence committed by coaches
		11	Lack of awareness of what educators should have
		13	Poor working conditions and treatment of coaches
	Player	1	Lack of basic knowledge about team sports
		2	Lack of basic manners in team sports
		3	Decrease in teamwork due to individual egoism
		6	Awareness of prioritizing parents’ opinions over those of coaches
	Parent	7	Parental involvement in training
		8	Parental involvement in hiring coaches
		10	Parental involvement in player management
		12	Parental involvement in school operation
		14	Parental involvement in game participation
Minority opinions		Not applicable	

**Table 9:** Results of 3rd Delphi survey.

Category	Derived items	Mean	CVR	Stability	Convergence	Agreement
Coach	1. Lack of leadership capacity of coaches	4.33	0.78	0.15	0.5	0.75
	2. Lack of leadership capacity of coaches in solving university entrance exams	4.78	1.00	0.09	0.5	0.8
	3. Student violence committed by coaches	4.33	1.00	0.11	0	0.8
	4. Lack of awareness of what educators should have	4.44	0.78	0.15		
	5. Poor working conditions and treatment of coaches	4.67	1.00	0.1	0.5	0.8
Player	6. Lack of basic knowledge about team sports	4.67	1.00	0.1	0.5	0.8
	7. Lack of basic manners in team sports	4.56	1.00	0.11	0.5	0.8
	8. Decrease in teamwork due to individual egoism	4.78	1.00	0.09	0.5	0.8
	9. Awareness of prioritizing parents’ opinions over those of coaches	4.56	1.00	0.11	0.5	0.8
Parent	10. Parental involvement in training	4.89	1.00	0.06	0.5	1
	11. Parental involvement in hiring coaches	5	1.00	0	0	1
	12. Parental involvement in player management	4.78	1.00	0.09	0.5	1
	13. Parental involvement in school operation	4.56	1.00	0.11	0.5	1
	14. Parental involvement in game participation	5	1.00	0	0	1

However, contrary to expectations, the Ministry of Culture, Sports and Tourism announced the abolition of the National Junior Sports Festival, which had served as an entry point for training elite athletes in 2019, but it was not implemented due to public opposition (3, 4), and the disbandment of school teams, which can be seen as the foundation of elite sports, continues from elementary school to high school and even university due to the decrease in the school-age population caused by the low birth (5). This phenomenon applies to all sports operated in Korea, but it is occurring rapidly in ice hockey, threatening the foundation of ice hockey.

The current status of elite ice hockey is as follows: As of 2014, there were a total of 15 teams, including 8 high schools, 5 universities, and 2 works teams. As of 2024, however, the number was reduced to 11, including 5 high schools, 4 universities, and 1 work team each for male and female SportsGyeongHyang (6). In addition, Jungdong High School with 78 years of history announced the disbandment of its ice hockey team, and Kyunghee University, which was founded in 1957, is barely surviving, maintaining the threat of continuous team disbandment (7, 8).

The urgency and importance of this phenomenon is that the tournament system for the competition cannot be operated. In other words, it means that ice hockey in Korea will disappear. Professional teams are already unable to hold league matches, and even college teams are facing this threat. Fortunately, the Korea Ice Hockey Association has been operating league matches by integrating college and professional teams since 2016 (9), but this is a temporary measure and could ultimately be a self-inflicted wound that could cause the meaning of existence of college and professional teams to decline. to summarize the problem, the rapid decline in the school-age population and the dissolution of college and professional teams are simultaneously being faced, and despite the current ongoing collapse of the system, neither the institutions that manage domestic ice hockey nor the academic community are paying attention to or researching this issue, which is why the urgency and importance of this study are emphasized.

Previous studies related to ice hockey are as follows: 195 studies were found based on the Korean Citation Index. However, it was found that they were mainly about technical analysis, injury and prevention, and physiological analysis of ice hockey. In particular, while studies on technical analysis were mainly conducted, it was found that one study on “Research Trends on Ice Hockey in Korean Physical Education Academia” and research on the environment of ice hockey clubs classified as sports for all was conducted by Kang (10), Park (11), and Lee (12). However, it was found that there are few studies related to elite sports, i.e., professional sports. In addition to this academic environment, the Ministry of Culture, Sports and Tourism, an organization related to ice hockey, has stated that there is no system to prevent the disbandment of university ice hockey teams, and the Korea University Sports Federation and the Korea Ice Hockey Association have stated that they have no authority. The schools, which are actually responsible for the operation, showed conflicting positions, that they are disbanding the team in accordance with the Ministry of Education's guidelines and that there was no organization willing to help (13).

It also attracted a lot of attention as it was selected as a unitary inter-Korean team at the 2018 Pyeongchang Winter Olympics. It seemed to be an issue in various directions as political issues between the South and the North and the composition of ice hockey team players were highlighted as social issues, but it ended up as a temporary phenomenon (14).

As a result, Suwon City Hall, which was the first professional women's ice hockey team formed for the Pyeongchang Olympics, is also discussing its transfer to the Korea Sports Promotion Foundation due to financial burden and insufficient game performance (15). showing that the ice hockey environment is gradually being driven into crisis.

It is noteworthy that the problems of ice hockey are not limited to Korea. Mccarver et al. (16) analyzed how parents in the United States interpret moral and immoral behavior in youth ice hockey, and found that the standards of hockey blur parents’ moral awareness and change into excessive conformity to sports ethics. This suggests that social moral values are given priority over values in a sports environment centered on athletic performance. In other words, it can be seen as a study that emphasizes the importance of the role of parents. Lemoyne and Tardif (17) argued that smooth communication between parents and coaches in Canadian ice hockey has a positive effect on creating a sports environment, so ice hockey associations and administrators should consider the preferences of parents and coaches when introducing programs. This was not identified as a problem, but it is not insufficient as a measure of how much influence coaches and parents have on ice hockey. In addition, research results have confirmed that parents who pursue stressful and authoritarian parenting styles in Finland do not hesitate to break the norms, and that the players of those parents also do not follow the norms (18). As the United States, Canada, and Finland described above are ice hockey powerhouses and active in the sport, it can be considered meaningful to explore research targeting those countries.

Therefore, the topic of this study, the analysis and examination of environmental problems with elite ice hockey and the measures for improvement, can be used as basic data for the development of the hard-won sport of ice hockey, and it can be also expected to be used as a basis for follow-up research. Thus, the researchers attempt to conduct the study in depth. The research questions set for systematic research are as follows. The re-search questions for systematic research are as follows:

First, what is the current status of elite ice hockey?
Second, why is the team disbandment occurring in elite ice hockey?
Third, what are the measures to improve the team disbandment in elite ice hockey?

## 2 Research methods

### 2.1 Research design

The method for deriving the results of this study is the “Delphi technique”, which is widely used as a technique for predicting the future. With a format for statistically ex-pressing opinions based on expertise in a specific field, the Delphi technique reflects the characteristics of qualitative and quantitative research by using questionnaires and inter-views with experts. In this study, the Delphi was conducted in three rounds as presented by Lawshe (19), and Seo and Lee (20).

### 2.2 Composition of the expert group

As the Delphi technique has statistical characteristics, but the research is conducted in an interview format, the expertise of the experts has a direct impact (21). Also, in order to minimize the reflection of this researcher's subjectivity, a separate group of experts who can check it was formed and given the role of intensive discussion on the research process and the derived research results. This measure is a procedure for securing the validity and reliability of this research and internal/external criticism. The group of experts formed consists of a total of three people: one professor from the Department of Physical Education and two doctoral-level researchers majoring in sports industry and management. Interventionary studies involving animals or humans, and other studies that require ethical approval, must list the authority that provided approval and the corresponding ethical approval code. The details are as in Table 1.

### 2.3 Delphi technique

#### 2.3.1 Literature review

A literature review was conducted to derive results for the first research question. The results of the literature review were used to secure the consistency of the research and conduct interviews with research participants. For the literature review, we used an electronic literature platform provided online, and selected, analyzed and used only data with clear sources such as Google Scholar and the Korean Citation Index (KCI) because the objectivity of the data is important (18). Search terms similar to specific words such as ice hockey, school, professional sports, and elite sports were used in the review, and public data was used such as media reports because there are relatively a few research materials in the academic world. The studies finally selected include 4 out of 144 academic studies, 1 out of 15 doctoral dissertations, and 27 press and media press releases.

#### 2.3.2 Literature review

The composition of the research participants was secured through convenience sampling, and the criteria for verifying expertise presented by the previously formed expert group were applied to the research participant setting. The three detailed criteria are as follows: A former elite ice hockey player, a person with more than 10 years of experience in coaching elite ice hockey players, and a person with a clear understanding of the current state of ice hockey. Based on the criteria, nine research participants were finally selected throughout the process, and only the minimum information was recorded as the Delphi technique emphasizes anonymity. The details are as in Table 2.

#### 2.3.3 Research tools and data analysis

The Delphi technique was conducted using a questionnaire and was implemented using a combination of online and offline methods. The “Google Online Survey” platform was used for online, while a separate meeting date was coordinated with the research participants for offline. The survey was conducted a total of three times from January 2024 to January 2025.

Round 1 of the Delphi technique was conducted using a semi-structured questionnaire, and Round 2 to Round 3 using a structured questionnaire. Round 2 was divided into majority and minority opinions based on the content structured through the inductive analysis of the findings from Round 1. Majority and minority opinions were divided based on more than 80% of research participants. Round 3 was a stage to statistically verify the consensus of the research participants, and CVR values were derived (21). The details are as in Table 3.

#### 2.3.4 Validity and reliability

The validity items of this study were composed based on expert verification, content validity ratio (CVR), stability, convergence, and agreement. Since there is a difference in the standard value for each item, it was set accordingly. Content validity ratio (CVR) was set to 0.78, the minimum value of 9 research participants suggested by Lawshe (19). and stability was set to 0.5 or less as a response consistency verification value. However, 0.6–0.8 was set as the acceptable range as it can be considered relatively stable. Convergence was set to 0.5 or less, and agreement to 0.75 or more (20).

## 3 Results

### 3.1 Analysis of the current state of elite Ice hockey

#### 3.1.1 Status of schools operating elite ice hockey and players

As a result of the first research question of this study, “What is the current status of elite ice hockey?”, the schools operating elite ice hockey and the number of players are as follows:

There are seven middle schools, six of which are male teams and one is female teams, while there are five high schools, all of which are male teams, and Jungdong High School is scheduled to be disbanded in 2025. There are four universities in operation, and all are male teams. It was found that HL Anyang is operated as a male team and Suwon City Hall as a female team among works teams. The details are as in Table 4.

In the registration status of elite ice hockey players, except for Namchuncheon Girls' Middle School, middle schools have secured at least 15 players, which is the minimum number of players required for ice hockey, and Kyunghee Middle School has the most players. It was found that high schools have more than 20 players, except for Jungdong High School, which is scheduled to be disbanded in 2025. While Korea University, Kwangwoon University, and Yonsei University have more than 25 players, Kyunghee University has the minimum number of players, 15. This means that the Kyunghee University ice hockey team is also at risk of disbanding, so it is trying to maintain its existence by forming a minimum number of members rather than operating actively. Among works teams, HL Anyang has 23 players and Suwon City Hall has 12 players, so only HL An-yang can participate in the regular league. The details are as in Tables 4, 5.

In addition to the currently operating school teams, we also examined the status of elite ice hockey teams that were disbanded within the past 10 years. A total of four middle schools were disbanded: Gyeongmin Middle School, Gwangseong Middle School, Boseong Middle School, and Yeonseong Middle School, while a total of three high schools were disbanded: Shinsong High School, Seondeok High School, and Boseong High School. Hanyang University was disbanded in 2021, and a total of three works teams were dis-banded: Daemyung Killer Whales, Korea Armed Forces Athletic Corps Sangmu, and High1. The common characteristic derived from the analysis of 11 disbanded teams is that the disbandment periods are concentrated in 2015–2016, 2019, and 2021–2023. There-fore, we analyzed what issues occurred during those periods, and the results are as fol-lows:

It was found that Gyeongmin Middle School, located in Uijeongbu-si, Gyeonggi-do had internal friction among the parents, which led to the disbandment of the ice hockey team, as the parents of the school voted to dismiss the coach at a general meeting regarding a fee increase proposed by the ice hockey coach, and the coach at the time as requested by the parents was rejected as not meeting the qualifications (22). In contrast, the ice hockey teams of Yeonseong Middle School and Shinsong High School, located in Incheon, and Seondeok High School, located in Dobong-gu, Seoul, were disbanded due to difficulties in recruiting players, indicating that difficulties in recruitment and operation of players at the time affected the existence of elite ice hockey teams (23, 24). The details are as in Table 6.

The Korea Armed Forces Athletic Corps, which was disbanded in 2019, had been disbanded in 2000, but was re-established in 2012 with the 2018 Pyeongchang Winter Olympics as an opportunity. However, it was disbanded after a temporary period of operation until the first half of 2019, which was an example of the government using sports for political purposes. At the time, the national team's demand to the government for a unified women's ice hockey team between the two Koreas was to maintain the Korea Armed Forces Athletic Corps ice hockey team, and the government promised support, but no follow-up measures were taken after the goal was achieved (25, 26).

In 2021, the coach at Boseong High School was found to have assaulted and abused students and engaged in financial transactions with parents, and the coach and some of the parents were sent to the prosecution. This result led to controversy over the school's mismanagement, which served as an opportunity to lose the duty and necessity for the ice hockey team, and the disbandment process began (27). Hanyang University announced the reason for disbanding the team as “difficulties in operation and management”, which can be seen as an extension of the disbandment of the gymnastics, track and field, and judo teams in 2015. This shows the reality of university sports with unpopular events, which expected to worsen further (28, 29).

The works teams Daemyung Killer Whales and High1 were disbanded for reasons different from those of the middle and high schools mentioned above. In December 2019, the emergence of COVID-19 and the pandemic caused economic damage to the whole world as well as Korea, and companies were at the crossroads of management aggravation and existence. In this situation, operating a sports team that requires a lot of invest-ment became a huge burden for companies. As games were not held due to the ban on gatherings, the goal was lost, leading to the disbandment of the team (30, 31).

In summarizing the current status of elite ice hockey, it can be interpreted that the closed culture that has been chronically appearing in the sports field and the social phenomena that appear externally are complexly having a negative impact on elite ice hockey, and the fact that the number of disbanded teams is more than 60% of the teams currently in operation means that the future of elite ice hockey is seriously uncertain. Therefore, in order to improve this situation, it is necessary to identify the main problems, and we would like to suggest the measures for improvement through the Delphi technique.

### 3.2 Analysis of the causes of the disbandment of elite Ice hockey teams

As a result of the second question of this study, “why is the team disbandment occur-ring in elite ice hockey?”, the causes of the disbandment of elite ice hockey teams, i.e., the problems, were summarized as follows through the Delphi 3 stages.

#### 3.2.1 Results of the 1st Delphi survey

The 1st Delphi survey was conducted based on analysis data on the current status of elite ice hockey, and 14 items were derived from the analysis of the survey contents. The details are as in Table 7.

#### 3.2.2 Results of 2nd Delphi survey

The 2nd Delphi survey was conducted with 14 items derived from the results of the 1st Delphi survey. The results of the 2nd Delphi survey did not have minority opinions and were composed only of majority opinions. The criteria for distinguishing between minority and majority opinions were set as whether more than 80% of the official research participants in this study agreed. The 14 items composed of majority opinions were set as categories of coaches, players, and parents through inductive analysis, and were com-posed of 5 items for the coach category, 4 items for the player category, and 5 items for the parent category. The details are as in Table 8.

#### 3.2.3 Results of 3rd Delphi survey

Details on this are in Figure 1. The 3rd Delphi survey, the final stage of this study, constructed factor output items for importance and consensus among experts as content validity ratio (CVR), stability, convergence, and agreement. The criteria for each factor were set as content validity ratio (CVR) of 0.75 or higher (19), stability of 0.8 or lower, convergence of 0.5 or lower, and agreement of 0.75 or higher (20). The items that met the criteria for each category are as follows: Among the five items in the leader category, the item “lack of awareness what educators should have” did not meet the convergence or agreement, while the player and parent categories met all items. Since the consensus of expertise in the Delphi technique is considered most important, if even one of the CVR, stability, convergence, and agreement was not met, this researcher excluded it after determining that expert opinions did not agree. The details are as in Table 9.

## 4 Discussion

The purpose of this study is to deeply analyze the phenomenon of team disbandment in elite ice hockey and to suggest ways to improve it. Therefore, based on the research results found through the Delphi 3 rounds and the data from previous studies and clear sources, the discussions and measures for improvement are described as follows:

### 4.1 Coaches

The sub-categories of the leaders constructed through Delphi, such as “Lack of leadership capacity of coaches in solving university entrance exams”, “Student violence com-mitted by coaches', and “Poor working conditions and treatment of coaches”, can be de-fined as problems that are contributing to the disbandment of elite ice hockey teams. Therefore, the related discussions and measures for improvement are as follows:

First, the fact that the leaders are responsible for disbandment of the elite ice hockey team means that they should reflect on and make efforts to improve as experts in the field and major in ice hockey. In particular, the coaches working in the elite ice hockey field have a duty to lay the foundation for their juniors to work in a better environment and demonstrate their expertise. Those involved in elite ice hockey need to reflect on them-selves to see if such efforts are being made.

However, this does not mean that the environment for coaches is prosperous. They have the role of presenting a bright future to middle and high school students who have entered elite ice hockey. The bright future referred to herein would be “going on to a prestigious university.” That is why the process of going on to a middle school with excellent athletic performance and to a high school with the potential to go on to a prestigious university is established. In such an environment, a leader has a lot of responsibility and pressure. This is because athletic performance is the leader's capacity, and they can re-main as good teachers through excellent admission rate to prestigious universities.

However, the number of students who can go on to prestigious universities such as Korea University and Yonsei University will be less than 30 per year, even if estimated broadly. Given that there are 102 high school athletes as of 2024, this means that less than 30% of them can go on to prestigious universities. Moreover, in the case of sports operated as teams, vacancies are filled depending on the position, so it would be reasonable to assume that the possibility of advancement is even lower. This kind of environment exposes coaches to wrong choices such as child abuse, and teams that fail to show their performance frequently change coaches due to low treatment of coaches, which leads to a de-cline in performance, repeating this cycle (32). As a result, if the players cannot go on to the university they want or as ice hockey players, the ice hockey they worked hard for in middle and high school will be meaningless, and if the coaches are not financially compensated, the team will have no choice but to disband. From a longer term perspective, university and works teams will also have a justification for disbandment due to a reduction in their base.

Therefore, in order to improve the disbandment of elite ice hockey teams, the treatment of coaches must be improved most urgently. Although this point has been suggested in various previous studies, there are clear limitations to the improvements in the treatment of coaches that can be provided at the school level. First, there is no related basis, that is, no legal system, and no standards have been established (33–35). So, in order to realistically improve treatment, an organization or group that can officially represent the position of coaches must be organized to create a pivot for coaches. In another form, establishing a certification agency for improving professionalism may be an alternative (36).

An institution or organization like a pivot is required because if you want to protect the rights and interests of leaders and demand improvements, a united opinion is essential. In a similar way, the necessity can be considered in the legal definition of a labor union as “an organization or federation of organizations organized by workers to voluntarily unite and maintain working conditions and improve the economic and social status of workers” (37). The key point of this plan is to emphasize that the improvement of treatment or environment of leaders can begin only when leaders demonstrate a movement for change.

In addition to improving the coaches' environment described above, coaches should make efforts to strengthen their leadership. In this study, the reasons why this phenomenon is occurring were divided into categories of coaches, players, and parents. In other words, since leaders are also focusing on this phenomenon, it can be seen as proof that they are not fulfilling their roles in their jobs, so coaches who are responsible for and operating the team need to strengthen their leadership to stabilize the team.

According to Yin et al. (38), a leader with conflict management style competency can provide a free communication atmosphere and provide the desire for expression among team members and can promote the team's innovative performance. In particular, since a cooperative conflict management style can be effective in situations where the team atmosphere is not good, it will be helpful in improving the environment currently facing elite ice hockey teams. Leadership is divided into various types, and transformational leadership has also been shown to have a significant impact on team cohesion (39). Specifically, it is necessary to approach the role of leadership, necessary knowledge, skills, and experience in the field-centered sports field (40).

It should also be considered that the relevance of the mindfulness interaction map suggested by Liu (41), such as team guidance, team creativity, and team cohesion, in sports teams can be utilized in a similar form to leadership. Such an approach and development in sports teams can serve as an opportunity to develop other areas such as fostering team culture, determining roles, identifying and selecting, developing and supporting, and evaluating and strengthening while more smoothly establishing the relationship between theory and practice (42). Previous studies have focused on the role of leadership in facilitating smooth interaction within a team. This is important in terms of theoretical research or learning capacity, but when considering the position and capacity of leaders in the field, it can be seen that leadership plays a major role (43).

### 4.2 Players

The sub-categories of players constructed through Delphi, such as “Lack of basic knowledge about team sports”, “Lack of basic manners in team sports”, “Decrease in teamwork due to individual egoism”, and “Awareness of prioritizing parents” opinions over those of coaches', can be defined as problems that are contributing to the disbandment of elite ice hockey teams. Therefore, the discussions and measures for improvement are as follows:

In elite ice hockey teams, players are the main characters who play the game. Both coaches and parents have a valid influence on the progress of the game, but without players, the game cannot go on, and players are in a position where they have to do their best more than anyone else as they are playing the game for their future. However, are they re-ally doing their best now? In team sports, not individual sports, individual ability is important, but teamwork is more important, but it is appropriate to say that current elite ice hockey players are “focusing on expressing their individual ability.” All the experts participating in this study commonly suggested that players prioritize satisfying their personal desires over the team winning, and that this aspect is getting worse.

Regarding this, Yevchenko et al. (44) reported to the academic world that although the evaluation of an individual's tendencies has various correlations, players who are aggressive and hostile have a negative impact on team cohesion, and the research results that the role of players who show team performance and team skills in the evaluation of sports events has a significant impact, and the research results that the degree of attachment within the team and the resulting relationship affect the overall part of the team are sufficient research results to support the findings presented in this study (45, 46).

Based on the problems described above and previous studies, if we analyze the core factors of the problem, it can be seen as the lack of basic awareness as a player. The problem described above is the lack of basic knowledge as a player, but the more serious problem is the lack of awareness. It is natural that there are differences because not all players can have the same ability even in team sports. If on the same team, they should rely on each other and respect each other to overcome together. This can also be found in the Olympic spirit. The Olympic spirit is “people from all over the world come together, respect each other, and practice tolerance and mutual understanding” (47). In other words, it is reasonable to assume that current elite ice hockey players are not aware of the virtues that sportspeople should have as a basic requirement.

This is an important problem that occurs in team sports as well as ice hockey. Previous studies have shown that individual game management has a significant effect on team performance, and that it affects not only team performance but also individual performance (48). The factor of efficient environment includes promoting friendship among team members, and the enjoyment factor of the team itself is a composition focusing on friendship, that is, the degree of friendship among team members affects athletic performance (49). Since team athletic performance exists separately from individual athletic performance in each sport, it has been argued that players should understand and train for team athletic performance (50).

However, despite such prior research or education of coaches, the reason why such problems persist is because players are prioritizing the opinions of their parents over those of their coaches when it comes to the progress of the game. In other words, there is a lack of basic “trust” among players, and “antisocial behavior” is being displayed due to a lack of commitment to the team (51). As the fundamental root of the lack of teamwork and lack of knowledge and awareness of team sports, which are the problems presented by this researcher, begins here, the influence of parents cannot be ruled out because the players are minors, but the players themselves must make their own efforts to be players. The problems and improvements for parents are discussed in the next chapter:Additional Requirements.

For additional requirements for specific article types and further information please refer to “Article types” on every Frontiers journal page.

### 4.3 Parents

The sub-categories of parents constructed through Delphi, such as “Parental involvement in training”, “Parental involvement in hiring coaches”, “Parental involvement in player management”, “Parental involvement in school operation”, and “Parental involvement in game participation”, can be defined as problems that are contributing to the disbandment of elite ice hockey teams. Therefore, the discussion and measures for improvement are as follows:

In the above, the problems of coaches and players and measures for improvement were presented. However, this study emphasizes that the fundamental problem lies with the parents. Until now, except for universities and works teams, parents are commonly involved in the disbandment of elite ice hockey teams. Parents participated in the committee for the discussion of disbandment and made the final decision on disbandment, and in some cases, parents voluntarily provided supplementary support through fund-raising as the treatment of coaches was poor. This clearly started as a voluntary act of parents for the players and coaches, but later changed into an act of so-called “gap-jil” (abuse of power) in which parents take the initiative over the coaches. In fact, parents' gap-jin over coaches, that is, anti-humanism and inhumanity, occurs frequently (52), and this culture is strongly reflected in ice hockey, unlike other sports.

The disbandment of the Gyeongmin Middle School ice hockey team can be regarded as a representative example. The discord among parents led to the disbandment of the school ice hockey team, and even at Jungdong High School, which boasts a 78-year history of ice hockey, some parents raised issues such as child abuse and violation of the Anti-Bribery Act, and the school decided to disband the team, citing damage to the reputation as an excuse. More than 90% of schools that operate elite ice hockey teams are private schools, and the school has the authority to establish and disband the team, so disbandment can be easier than expected. Regardless of right or wrong, these results prove that the parents' behavior played a causal role in the disbandment of elite ice hockey teams.

Not stop at school management and coach hiring, parents are also deeply involved in player management, training, and games. Players' lack of basic knowledge of team sports, manners, or respect that this researcher suggested earlier also arises when parents’ thoughts are transferred to the players. Even if the players are middle and high school students who have physically grown and have passed puberty, their parents' role and influence are absolutely important until they become adults and (53), That is why the players recognize and act on the opinions of their parents rather than those of their coaches.

Unfortunately, previous studies have confirmed that the same problem is occurring not only in Korea but also around the world. Danioni and Barni (54) found that the mother's status among parents is a major variable in predicting antisocial behavior in adolescent athletes, and argued that the higher the frequency of an atmosphere focused only on performance, the higher the antisocial behavior. In addition, it has been confirmed that studies have been reported in academic circles that the stronger the parents' perception of social identity, the higher the possibility of antisocial behavior in adolescents (55). In addition to the fact that parents have a significant influence as a cause of antisocial behavior in sports, research results have also confirmed that problems are occurring in ice hockey sports in other countries. The results showing that parents are the cause of game management that can be seen as unsportsmanlike behavior can be considered to be an inherent problem of ice hockey (56).

The implications of the research results that previous studies commonly show emphasize that parents themselves should act to prevent antisocial behavior in players, as the influence of parents has a direct and significant effect on adolescents (57). This is consistent with the opinions commonly presented by the experts who participated in this study, so it can be considered the most important part of this study.

Therefore, this researcher emphasizes that a culture in which parents trust coaches and players and refrain from excessive involvement should be spread in order to form a healthy elite ice hockey culture. If parents continue to act as coaches and players as they do now, elite ice hockey will not be recognized as a sport in Korea.

## 5 Conclusions

The purpose of this study is to analyze the team disbandment in elite ice hockey and to suggest the measures for improvement. The research method used to achieve the purpose of the study was the Delphi technique, which was conducted in three rounds to de-rive detailed items, distinguish majority and minority opinions, and draw CVR, stability, convergence, and agreement. Based on the research results, three categories were com-posed: coaches, players, and parents. The detailed items of the coach category were com-posed of “Lack of leadership capacity of coaches in solving university entrance exams”, “Student violence committed by coaches”, and “Poor working conditions and treatment of coaches”. The detailed items of the player category were composed of “Lack of basic knowledge about team sports”, “Lack of basic manners in team sports”, “Decrease in teamwork due to individual egoism”, and “Awareness of prioritizing parents” opinions over those of coaches'. The detailed structure of the parent category was composed of “Pa-rental involvement in training”, “Parental involvement in hiring coaches”, “Parental involvement in player management”, “Parental involvement in school operation”, and “Pa-rental involvement in game participation”.

This study focused on analyzing the phenomenon of disbanding elite ice hockey teams in Korea, so it mainly utilized literature and media reports published in Korea. In the discussion for comparative analysis of the research results, papers of SCI level or higher were utilized, but since it was a priority to identify problems occurring only in Korea, it was decided to mainly utilize domestic literature.

While conducting this study, this researcher could not imagine a bright future for elite ice hockey in Korea. Elite ice hockey is continuously decreasing because the population of ice hockey players at the level of sports for all is continuously increasing, but sports for all and elite sports systems are disconnected. The reason why ice hockey is developing as sports for all is as follows: Most elementary school students who learn ice hockey do not learn it to become elite ice hockey players, but because their ice hockey-related experience is recognized through qualitative evaluation when immigrating to the United States or Canada or entering international schools. Like golf, ice hockey is recognized as a sport that requires a lot of money, and in reality, a lot of money is invested in entering and maintaining. Therefore, parents are financially well-off, showing the characteristic of freely traveling not only domestically but also internationally. However, only the com-plaints, dissatisfaction, or unconditional acceptance for this environment cannot be the answer. Since improvements take a long time, but entry into the reduction or discontinuance stage is rapid, a lot of interest and effort from ice hockey players will be required in order to preserve the system that has been built until now. We hope that the results of this study will be used as basic data for the efforts.

## Data Availability

The datasets presented in this study can be found in online repositories. The names of the repository/repositories and accession number(s) can be found in the article/Supplementary Material.
